# Supplementary material for: Random Allelic Expression in Inherited Retinal Disease Genes
Source: Curr Issues Mol Biol. 2023 Dec 13;45(12):10018–25. doi: 10.3390/cimb45120625 (PMC10742332; doi:10.3390/cimb45120625)
Supplement: Supplementary file 1 [file cimb-45-00625-s001.zip › Table S1. List of genes from RetNet that exhibit biallelic expression (BAE).pdf]

Table S1. List of genes from RetNet that exhibit biallelic expression (BAE).

BAE Genes	ABCA4
	ACBD5
	ADAM9
	ADGRV1
	ADIPOR1
	AGBL5
	AHR
	ARHGEF18
	ARL3
	ARL6
	ARSG
	ATF6
	ATXN7
	BBIP1
	BBS1
	BBS10
	BBS12
	BBS4
	BBS5
	BBS7
	BBS9
	BEST1
	C12orf65
	C1QTNF5
	C8orf37
	CA4
	CABP4
	CACNA2D4
	CCT2
	CDH3
	CEP164
	CEP19
	CEP250

	CEP290
	CEP78
	CERKL
	CLN3
	CLUAP1
	CNGA3
	CNGB1
	CRB1
	CSPP1
	CWC27
	DHDDS
	DHX38
	DRAM2
	DTHD1
	DYNC2H1
	ELOVL1
	ELOVL4
	EMC1
	EXOSC2
	EYS
	FAM161A
	FZD4
	GDF6
	GNPTG
	GRM6
	GUCY2D
	HARS
	HGSNAT
	HK1
	HMCN1
	HMX1
	IDH3B
	IFT172
	IFT27

	IFT81
	IMPG1
	IMPG2
	INVS
	ITM2B
	KCNV2
	KIAA1549
	KIF11
	KIF3B
	LCA5
	LRAT
	LRIT3
	LRP5
	LZTFL1
	MAK
	MFSD8
	MIEF1
	MKKS
	MKS1
	MVK
	NBAS
	NEK2
	NEUROD1
	NMNAT1
	NPHP1
	NR2F1
	NRL
	OPA1
	OPA3
	OPN1SW
	OTX2
	PANK2
	PAX2
	PCDH15

	PDE6A
	PDE6C
	PDZD7
	PEX2
	PEX7
	PLK4
	POC1B
	POC5
	POMGNT1
	PRPF3
	PRPF31
	PRPF6
	RAB28
	RD3
	RDH11
	RDH12
	RGR
	RGS9BP
	RIMS1
	RLBP1
	ROM1
	RP1
	RP1L1
	RP9
	RPE65
	RPGRIP1
	RPGRIP1L
	RTN4IP1
	SAMD11
	SDCCAG8
	SLC24A1
	SLC25A46
	SLC7A14
	SNRNP200

	SPATA7
	TEAD1
	TMEM126A
	TMEM216
	TMEM237
	TOPORS
	TRIM32
	TRNT1
	TTC8
	TTLL5
	TTPA
	TUBGCP4
	TUBGCP6
	TULP1
	UNC119
	USH1G
	WDR19
	ZNF423
