# Supplementary material for: Random Allelic Expression in Inherited Retinal Disease Genes
Source: Curr Issues Mol Biol. 2023 Dec 13;45(12):10018–25. doi: 10.3390/cimb45120625 (PMC10742332; doi:10.3390/cimb45120625)
Supplement: Supplementary file 1 [file cimb-45-00625-s001.zip › Table S2. Additional biologic processes significantly enriched for BAE.pdf]

Table S2. Additional biologic processes significantly enriched for biallelic expression (BAE) genes.

**Biallelic Expression, Significantly Enriched Biological Process**
fat-soluble vitamin metabolic process
centrosome cycle
smoothened signaling pathway
plasma membrane bounded cell projection assembly
fat cell differentiation
cell projection assembly
transport along microtubule
heart looping
response to light stimulus
determination of left/right symmetry
microtubule organizing center organization
left/right pattern formation
determination of bilateral symmetry
specification of symmetry
determination of heart left/right asymmetry
embryonic heart tube morphogenesis
retinoid metabolic process
protein-containing complex localization
diterpenoid metabolic process
regulation of smoothened signaling pathway
neural tube closure
cytoskeleton-dependent intracellular transport
tube closure
microtubule-based transport
spindle assembly
terpenoid metabolic process
primary neural tube formation
isoprenoid metabolic process
inner ear development
neural tube formation
ear development
response to radiation
tube formation
epithelial tube formation
organelle assembly
neural tube development
embryonic epithelial tube formation
establishment of vesicle localization
cell projection organization
plasma membrane bounded cell projection organization
embryonic organ morphogenesis

microtubule-based movement
microtubule-based process
regionalization
detection of stimulus
microtubule cytoskeleton organization
pattern specification process
embryonic organ development
epithelial tube morphogenesis
morphogenesis of an epithelium
embryonic morphogenesis
protein localization to organelle
response to abiotic stimulus
detection of stimulus involved in sensory perception
tissue morphogenesis
cellular component assembly
cellular component biogenesis
protein localization
cellular macromolecule localization
embryo development
neuron projection development
establishment of protein localization
epithelium development
cytoskeleton organization
macromolecule localization
head development
brain development
cellular localization
organelle organization
protein transport
intracellular transport
protein-containing complex assembly
anatomical structure morphogenesis
nitrogen compound transport
cellular component organization
cell development
response to external stimulus
cellular component organization or biogenesis
localization
organic substance transport
cell differentiation
cellular developmental process
establishment of localization
anatomical structure development
developmental process

transport
response to stimulus
cellular process
biological_process
immune response
Unclassified
